# Transcriptome Analysis Elucidates the Key Responses of Bryozoan *Fredericella sultana* during the Development of *Tetracapsuloides bryosalmonae* (Myxozoa)

**DOI:** 10.3390/ijms21165910

**Published:** 2020-08-17

**Authors:** Gokhlesh Kumar, Reinhard Ertl, Jerri L. Bartholomew, Mansour El-Matbouli

**Affiliations:** 1 Clinical Division of Fish Medicine, University of Veterinary Medicine, 1210 Vienna, Austria; Mansour.El-Matbouli@vetmeduni.ac.at; 2 VetCore Facility, University of Veterinary Medicine, 1210 Vienna, Austria; reinhard.ertl@vetmeduni.ac.at; 3 Department of Microbiology, Oregon State University, Corvallis, OR 97331-3804, USA; barthoje@oregonstate.edu

**Keywords:** RNA-seq analysis, gene expression, bryozoan response, zooid, myxozoan parasite

## Abstract

Bryozoans are sessile, filter-feeding, and colony-building invertebrate organisms. *Fredericella sultana* is a well known primary host of the myxozoan parasite *Tetracapsuloides bryosalmonae*. There have been no attempts to identify the cellular responses induced in *F. sultana* during the *T. bryosalmonae* development. We therefore performed transcriptome analysis with the aim of identifying candidate genes and biological pathways of *F. sultana* involved in the response to *T. bryosalmonae*. A total of 1166 differentially up- and downregulated genes were identified in the infected *F. sultana*. Gene ontology of biological processes of upregulated genes pointed to the involvement of the innate immune response, establishment of protein localization, and ribosome biogenesis, while the downregulated genes were involved in mitotic spindle assembly, viral entry into the host cell, and response to nitric oxide. Eukaryotic Initiation Factor 2 signaling was identified as a top canonical pathway and MYCN as a top upstream regulator in the differentially expressed genes. Our study provides the first transcriptional profiling data on the *F. sultana* zooid’s response to *T. bryosalmonae*. Pathways and upstream regulators help us to understand the complex interplay in the infected *F. sultana*. The results will facilitate the elucidation of innate immune mechanisms of bryozoan and will lay a foundation for further analyses on bryozoan-responsive candidate genes, which will be an important resource for the comparative analysis of gene expression in bryozoans.

## 1. Introduction

*Fredericella sultana* is a freshwater bryozoan and is found worldwide. It is typically colonial, consisting of up to several hundred connected individual zooids. Each zooid has its own independent tentacular lophophore used for suspension feeding [1]. *F. sultana* reproduces both asexually and sexually. The colonies propagate quickly during spring and form tubular and branching colonies that attach to submerged surfaces like pieces of wood and tree roots by summer. Over the winter period, they produce dormant seed-like stages called statoblasts [2]. Recent completion of a de novo transcriptome assembly of *F. sultana* provides insight into the unique cellular, metabolic, and catalytic processes of this bryozoan species [3].

*F. sultana* is the most common bryozoan host of *Tetracapsuloides bryosalmonae* (Myxozoa), the causative agent of proliferative kidney disease in salmonids. The cycle of the parasite alternates between covert and overt infection in bryozoans. The spores of *T. bryosalmonae* develop first as a covert infection (single-cell stages) within the bryozoan body wall. At the time of overt infection, multicellular sacs produce from single cell stages and multiply in the body cavity of the bryozoans [4,5]. Covert infections are widespread and persist throughout the year in bryozoans [6]. Infected bryozoan colonies release parasite spores into the surrounding water [7]. The spores enter the fish host through the gill epithelium, multiply in the vascular system, and reach the kidney as the main target organ. Further parasite development in that tissue causes an inflammatory response and damage to kidney tissues [8,9].

Due to being the primary host of an economically important fish parasite, *F. sultana* has become one of the most studied bryozoan species. A number of studies have investigated the ecology of the bryozoan in relation to its infection status [10]. *T. bryosalmonae* infection reduces utilization of food levels, growth rates, and reproduction in *F. sultana* [11]. Interactions between *F. sultana* and *T. bryosalmonae* are associated with multiple transmission pathways and host condition-dependent developmental cycling [12]. Now that we have a *F. sultana* transcriptome that identifies the unique characteristics of this organism [3], we can investigate how these genes are modulated in response to infection by *T. bryosalmonae*. Understanding the cellular triggers that occur when the bryozoan is infected with the parasite will allow us to elucidate molecular immunity and related components in the defense system of bryozoan. This will be a useful starting point to enhance our understanding of the mechanisms of immunity of bryozoan to *T. bryosalmonae* infection at the molecular level, by combining a variety of bioinformatic analyses for studies of host-parasite interactions, gene discovery, and novel antigen identification.

Understanding the dynamic interactions at the bryozoan-host interface can be achieved in part by characterizing bryozoan gene expression relative to *T. bryosalmonae* infection. Towards this end, we aimed to analyze transcriptomic changes in the zooids of *F. sultana* during the *T. bryosalmonae* development using high-throughput transcriptome sequencing. To study the molecular mechanism of *F. sultana*, we performed enrichment analysis and functional annotation of the differentially expressed genes (DEGs) involved in a plethora of biological roles in different immune system processes and pathways.

## 2. Results

### 2.1. RNA-Seq Analysis

To gain insight into the dynamic alterations in gene expression of *F. sultana* during parasite development, cDNA libraries were constructed for RNA-seq from infected and uninfected zooids. After data analysis and quality filtering of the sequencing data, we obtained a total of 274 million raw reads (paired) of the zooids transcriptome. We obtained a total of 250 million high-quality reads (paired) ranging from 37 to 44 million trimmed reads per sample after removal of low-quality reads and contaminating adapter sequences (Table 1).

**Figure 1:**
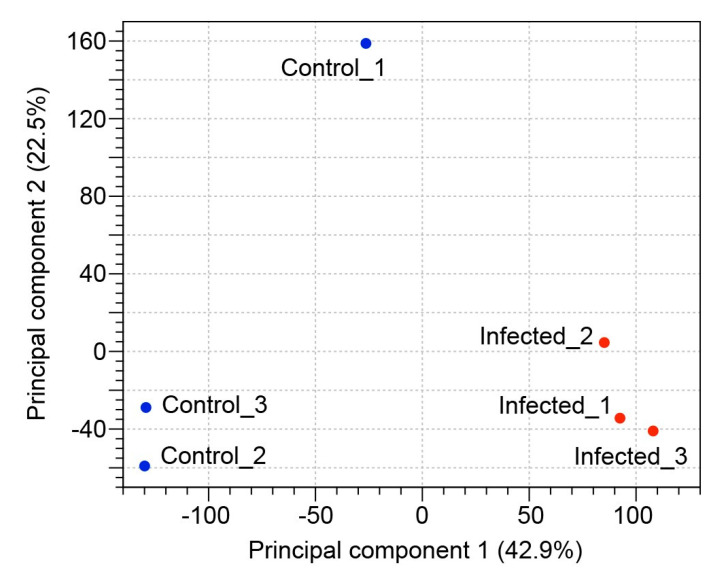
Principal component analysis. PCA plot of normalized RNA-seq expression values of infected and uninfected control replicate zooid samples.

**Figure 2:**
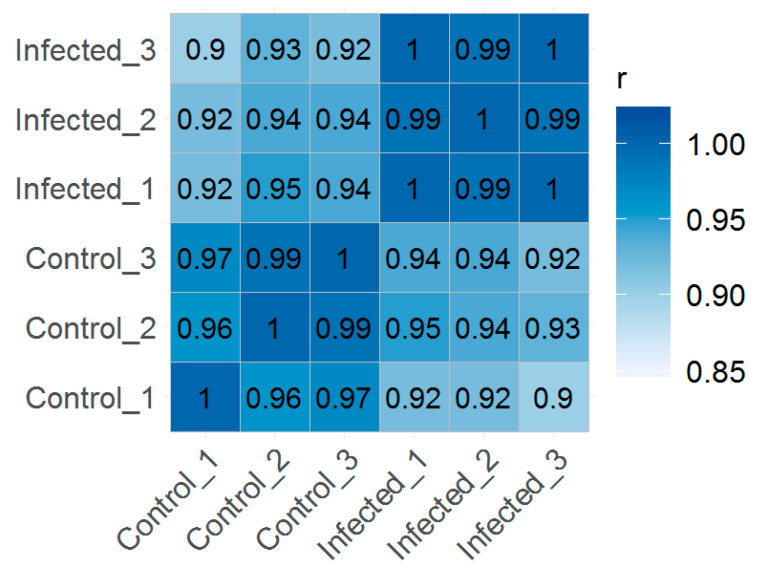
Global correlation analysis. Heatmap showing Pearson’s correlation coefficient (*r*) for TPM normalized values across samples, indicating positive correlation between biological replicates.

Based on the random clustering of the replicates in the principal component analysis (PCA) plot (Figure 1), we assume a high degree of uniformity among the replicates. The results observed in the PCA showed that the first principal component explained 42.9% of the total variation in our experimental data and clearly separated the infected group from uninfected control group. The correlation between transcripts per million (TPM) normalized values from replicate samples showed positive correlation between the samples ranged from 0.90 to 1.00, indicating that the sample repeatability was reliable (Figure 2).

**Figure 3:**
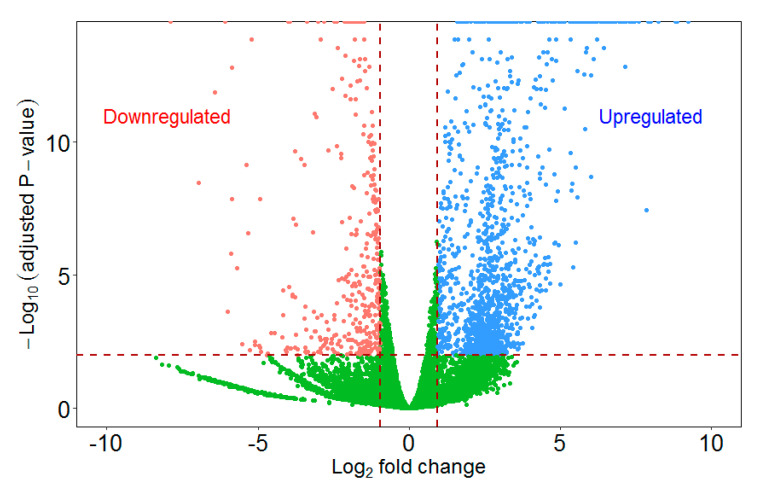
Volcano plot of differentially up- and downregulated genes in the infected zooids relative to the uninfected control zooids. A volcano plot shows the relationship between the *p*-values of a statistical test and the magnitude of the difference in expression values. On the y-axis the negative log_10_ *p*-values are plotted. On the x-axis the log_2_ values of the fold changes seen in whole transcriptome comparison.

We performed differential expression analysis to identify quantitative gene expression changes between infected and uninfected zooids. The statistical analysis revealed a total of 1166 differentially regulated genes (FDR adjusted *p*-value ≤ 0.01 and fold change ≥2 or ≤−2) in the infected zooids of *F. sultana* (Figure 3). Of these, 926 genes were upregulated, and 240 genes were downregulated in the infected zooids. The details of fold change and enriched gene ontology terms of all differentially regulated genes are presented in Tables S1 and S2, respectively.

**Figure 4:**
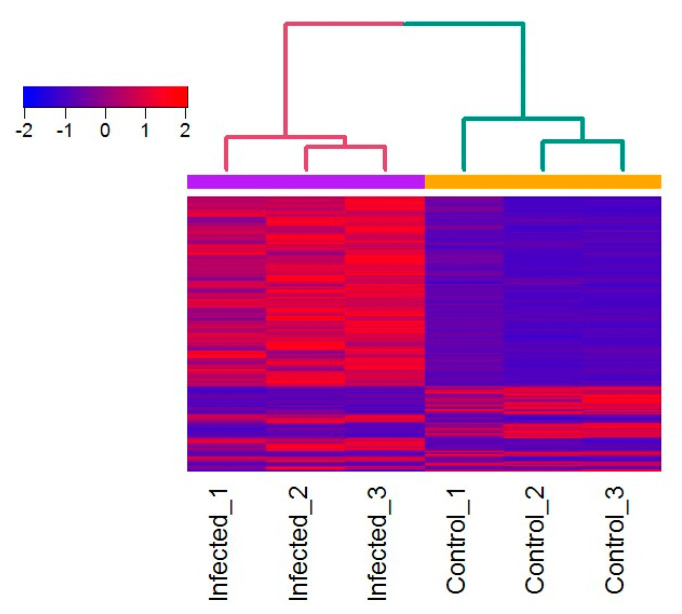
Heatmap showing the expression patterns of genes in the zooid samples. Heatmap of Z scaled RNA-seq normalized TPM values of 1166 differentially expressed transcripts (FDR adjusted *p*-value: ≤0.01) among the infected and uninfected control zooids. Red indicates upregulation; blue indicates downregulation, as indicated in the color key. Each row represents one gene and each column represents an individual zooid. Euclidean distance and complete linkage method was used for hierarchical clustering.

The heatmap displaying the broad patterns of gene expression (Figure 4) in the infected zooids highlighted a subset of genes that were activated in response to *T. bryosalmonae*.

### 2.2. GO Annotation

**Figure 5:**
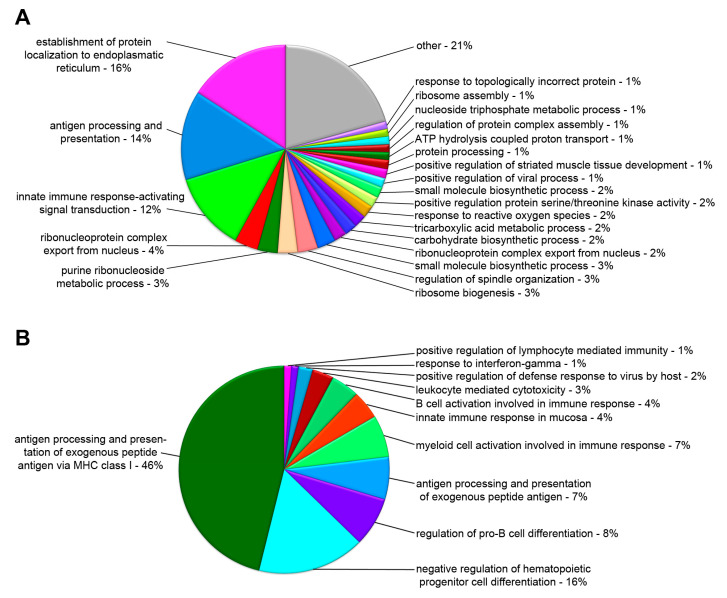
Percentage of significantly enriched Gene Ontology terms for the categories. Biological Process (**A**) and Immune System Process. (**B**) As identified by ClueGO. GO terms with similar functions were merged into groups based on the overlap of the associated genes. Groups were named after the most significantly enriched GO term.

To reveal the underlying cellular mechanisms associated with the DEGs, we performed GO-term enrichment analysis using the ClueGO v2.2.4 software, to detect and categorize over-represented GO-terms based on the annotations found in our gene set. Within the classification of biological process, all the differentially up- or downregulated genes were mainly associated with establishment of protein localization to endoplasmic reticulum, antigen processing and presentation, innate immune response, ribonucleoprotein complex, ribosome biogenesis, and several metabolic processes (Figure 5A). In terms of the immune system process, differentially up- or downregulated genes were enriched in antigen processing and presentation of exogenous peptide antigen via MHC class I, negative regulation of hematopoietic progenitor cell differentiation, regulation of pro-B cell differentiation, and myeloid cell activation (Figure 5B).

### 2.3. Canonical Pathways and Upstream Regulators

**Figure 6:**
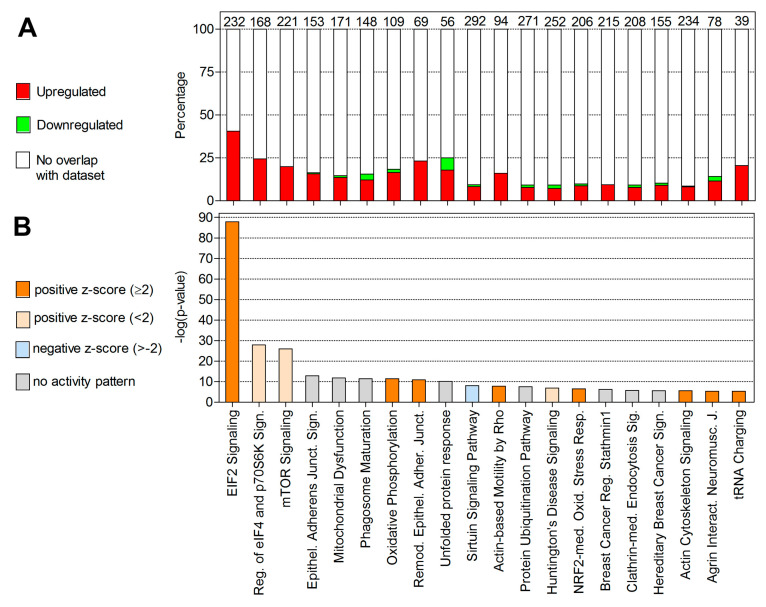
Top 20 most significantly enriched canonical pathways in the infected zooids as identified by Ingenuity Pathway Analysis. (**A**) Percentage of DEGs overlapping with molecules associated to the top 20 most significantly enriched canonical pathways. Colors represent up- (red) and downregulation (green). Numbers on top of the bars represent the total number of molecules associated to each pathway. (**B**) Bars represent the *p*-values of overlap (−log) of DEGs in the dataset with known pathway-associated molecules. Colors denote the predicted activation (z-score: ≥2) or inhibition (z-score ≤−2) state.

To understand the function of DEGs in different modules more broadly, we also performed canonical pathway enrichment analysis using Ingenuity Pathway Analysis (IPA) software. The top 20 enriched canonical pathways identified by IPA are shown in Figure 6. Additionally, Figure 6 shows the percentage of DEGs regulated relative to those present in the IPA knowledge base that are assigned to a given pathway and the direction of their regulation. The most significant pathways associated with the DEGs in our dataset are eukaryotic initiation factor 2 (EIF2) signaling, regulation of eIF4 and p70S6K signaling, mTOR signaling, epithelial adherens junction signaling, mitochondrial dysfunction, phagosome maturation, and oxidative phosphorylation. The details of enriched canonical pathways are presented in Table S3.

**Figure 7:**
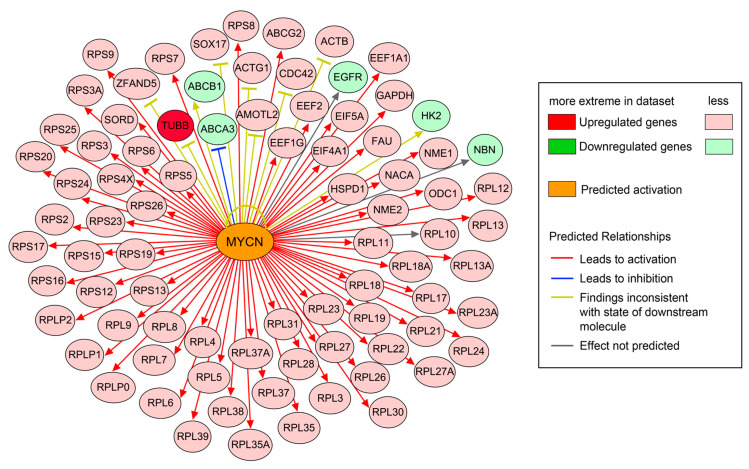
Effect of the upstream regulator molecule MYCN on 82 target genes found differentially expressed in the infected zooids. Molecules in red denote upregulation; molecules in green denote downregulation and orange denotes a predicted activation. Coloring of the lines denotes the predicted effects: activation (red), inhibition (blue), and not predicted (grey) on the target molecules or indicates inconsistent findings with the state of the downstream molecules (yellow).

Our objective here was to identify upstream regulators of DEGs that are associated and activated in *F. sultana* during parasite development using IPA software. Upstream regulator analysis identified 82 direct targets among the 1166 DEGs (Figure 7). The top upstream regulator identified by IPA was MYCN. Upstream regulator analysis predicts that the majority of target genes would be activated (or upregulated, as indicated by red arrows) by MYCN. A brief summary of IPA analysis of DEGs identified in the infected zooids is presented in Table 2 and details of upstream regulators are given in Table S4.

### 2.4. Validation of Transcriptional Regulation

**Figure 8:**
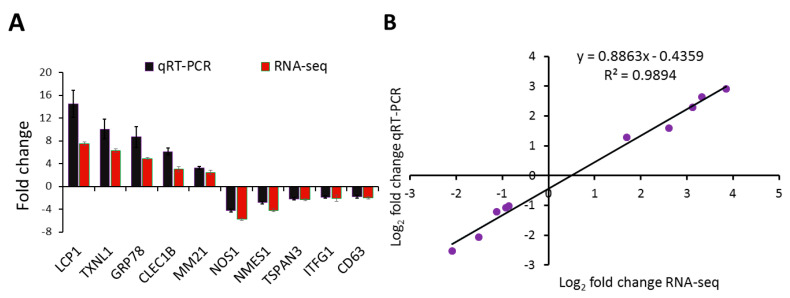
Validation of RNA-seq by qRT-PCR and linear regression plot between RNA-seq and qRT-PCR. (**A**) Five upregulated and five downregulated genes determined by differential gene expression analysis of RNA-seq data were selected for qRT-PCR analysis. The gene expression values are represented as relative fold change (mean ± SEM) of infected zooids (*n* = 6) compared to the uninfected control zooids group (*n* = 6). (**B**) The log_2_ fold change values for the RNA-seq and qRT-PCR are plotted along with the linear fit line showing a significant Pearson’s correlation coefficient *r* (8) = 0.995, *p* < 0.00001 and a coefficient of determination R^2^ = 0.989.

To validate the findings from RNA-seq, we performed quantitative real-time PCR for 10 genes of interest. As shown in Figure 8A, the relative transcript levels measured by qRT-PCR were consistent with the corresponding RNA-seq data obtained from the RNA-seq analysis. The correlation between RNA-seq and qRT-PCR data was performed using linear regression analysis. A significant positive correlation (*r* (8) = 0.994, *p* < 0.00001) between the log_2_ fold change values of RNA-seq and qRT-PCR confirms the consistency and reproducibility of the RNA-seq analysis (Figure 8B).

### 2.5. Data Availability

The raw RNA-seq reads have been deposited in the NCBI SRA database under the accession numbers: SRP198874 and BioProject ID PRJNA543711 for uninfected control zooids and SRP261017 and BioProject ID PRJNA631572 for infected zooids.

## 3. Discussion

The bryozoan *Fredericella sultana* is an aquatic invertebrate that acts as the definitive host for the malacosporean parasite *T. bryosalmonae*. This parasite causes proliferative kidney disease in salmonids in Europe and North America [13]. Nothing is known about the immune system of *F. sultana*. In this study, we have investigated the transcriptional response of *F. sultana* to *T. bryosalmonae* in order to illuminate the putative mechanism underlying the immune response during parasite development. Our results have shown that infected *F. sultana* induced EIF2 signaling, regulation of eIF4 and p70S6K signaling, mTOR signaling, epithelial adherens junction signaling, mitochondrial dysfunction, phagosome maturation, and oxidative phosphorylation pathways.

In invertebrates, the innate immune system is the main strategic defense mechanism to pathogens. Gene ontology analysis of *F. sultana* revealed that the majority of classifiable transcripts were products from genes that play roles in the innate immune system (Figure 5). This is perhaps not surprising as overt infection of the parasite is known to exert a pleiotropic effect on *F. sultana* growth [14]. We found DEGs related to antigen processing and presentation, myeloid cell and B cell activation, regulation of pro-B cell differentiation, and negative regulation of hematopoietic progenitor cell differentiation. We also found EIF2 signaling, regulation of eIF4 and p70S6K signaling, mTOR signaling, and phagosome maturation pathways in the infected zooids. The upregulated genes involved in the pathway were localized in the cytosolic ribosome and vacuole (Table S5). Accordingly, we found that the pathway related to ribosome is affected in the infected zooids (Figure 7). Enrichment of genes implicated in ribosome, translation, and nucleic acid binding in these clusters suggested that genes involved in basal cellular activity were expressed in bryozoans during parasite development. These data suggested that the signaling responses for the innate immune response and protein synthesis are major events in *F. sultana* during parasite development. As a large number of immune related genes were found to be concurrently upregulated upon parasite development, we focused this study on the molecular immune mechanism of *F. sultana* against *T. bryosalmonae* infection.

Lectins are carbohydrate-binding proteins that have a role in recognition at the cellular and molecular level. Lectins interact with pathogen-associated molecular patterns (PAMPs) to activate innate immunity [15]. In the present examination, four DEGs related to lectins, including lectin L6-like (2.61 fold), lactose-binding lectin l-2-like (2.89 fold), C-type lectin domain family 12 member B isoform X2 (3.02 fold), and fucolectin isoform X1 (3.03 fold) showed significantly upregulated expression in the infected *F. sultana*. However, other lectins such as galactose-specific lectin nattectin-like (−8.41 fold), C-type lectin 37Db-like (−12.99 fold), perlucin-like protein (−42.01 fold), and galectin 2 (−2.54 fold) were significantly downregulated in the infected *F. sultana*. Specific lectin-carbohydrate binding molecules at the parasite membrane and host–parasite interaction have been described earlier for myxozoans such as *T. bryosalmonae* and *Myxobolus cerebralis* [16,17]. Upregulation of lectins (4.97 to 12.43 log_2_ fold) was also observed in oligochaete *Branchiura sowerbyi* in response to myxozoan *Myxobolus cultus* infection [18]. The interaction between *T. bryosalmonae* and the rhamnose-binding lectin STL1 from the kidney of brown trout was identified by antibody-based protein purification followed by ESI-MS [19]. The identified upregulated lectins might be involved in *T. bryosalmonae* attachment and the subsequent activation of the immune response system of *F. sultana* to eliminate the parasite infection and maintain homeostasis. However, the roles of identified downregulated lectins need to be investigated using molecular characterization of lectins to provide further insight into the bryozoan’s immune response and downstream pathway activation.

We found that the expression of 20 ubiquitin related genes such as ubiquitin-like protein, polyubiquitin, SCF ubiquitin ligase, ubiquitin-conjugating enzyme E2, ubiquitin-40S ribosomal protein S27a, and ubiquitin-60S ribosomal protein L40 were significantly upregulated in the infected zooids. Ubiquitin-mediated proteolysis activates the innate immune system and motivates a variety of immune-related regulators from transcription to apoptosis [20]. A previous study reported that the ubiquitin-mediated proteolysis pathway was involved in annelid defense response against myxozoan *B. sowerbyi* [18]. This suggests that the ubiquitin-mediated proteolysis pathway was activated by bryozoans in response to *T. bryosalmonae* infection. To maintain the endogenous redox homeostasis, bryozoans must cope with the oxidative challenges caused by the host immune system against *T. bryosalmonae*.

Oxidative stress is an important process in the recognition and elimination of pathogens. The spores of *T. bryosalmonae* adhere at the inside of bryozoan peritoneal wall and they develop and float in the coelomic fluid of the body cavity of bryozoan [21]. Based on the KEGG database analysis, we found enrichment of DEGs in phagosome (23 DEGs), lysosome (14 DEGs), oxidative phosphorylation (12 DEGs), and peroxisome (6 DEGs) pathways. Phagosome pathway related genes such as calreticulin, ras-related proteins (Rab-5B, Rab-7a, and C3 botulinum toxin substrate 1-like), and toll-like receptors (TRL3, TLR4, and TRL 13) were upregulated in the infected zooids, suggesting that the response of bryozoans was conducive for the formation of phagolysosomes to clear *T. bryosalmonae*. Lysosome, peroxisome, and oxidative phosphorylation pathways genes like cathepsin B, lysosomal alpha-mannosidase, peroxiredoxin-5, superoxide dismutases (Fe and Cu-Zn), cytochrome c oxidase subunit V, and NADH dehydrogenase (ubiquinone) iron-sulfur proteins (2, 3, and 7) were upregulated in the infected zooids. Similarly, the upregulation of TLR13-like, cathepsin B, peroxiredoxin, and cytochrome c oxidase subunit 5B was observed in the kidney of brown trout in response to *T. bryosalmonae* [22,23]. The upregulation of phagosome and antioxidative pathways was also demonstrated in oligochaete worms in response to the myxozoan parasite *B. sowerbyi* [18]. Combined, these results suggest that myxozoan parasites induce innate immunity in their primary hosts. Overall, upregulation of DEGs related to these pathways activates innate immune defense processes that could protect bryozoans during the infection of *T. bryosalmonae*. However, some immune-related genes such as lysosomal protective protein-like (−4.37 fold), lysosome-associated membrane glycoprotein 1-like (−3.96 fold), macrophage mannose receptor 1-like (−58.36 fold), CD63 antigen (−2.04 fold), CD82 antigen-like (−4.10 fold), CD225/dispanin family protein (−2.18 fold), T-cell immunomodulatory protein (−2.19 fold), and TNF receptor-associated factor 2 isoform X1 (−2.93 fold) were downregulated in the infected zooids. Further study would be required to provide insight into the innate immune responses that function in bryozoan’s immune network and the relevant effector responses.

Numerous proteases that belong to the peptidase, hydrolase, and metalloprotease families were upregulated in the infected zooids. Cathepsins, a superfamily of hydrolytic enzymes enclosed within lysosomes, contribute to several physiological and immune response activities [24]. Here, the significantly upregulated expression of cathepsin L (6.38 fold) and cathepsin D (15.49 fold) in the infected bryozoans suggested an increased probability of reducing parasite infection in bryozoans. Carboxypeptidases are another type of peptidases that cleave and release free amino acids one by one from the C-terminus of the peptide chain and play many important functions in innate immunity [25]. Here, a total of four carboxypeptidases (CPVL (6.40 fold), CPN2 (2.85 fold), CPB (3.32 fold), and CPE (6.03 fold)) were upregulated in the infected zooids. Carboxypeptidases may have a gatekeeper function in the signaling transduction factors in regulating innate immunity and thus play a defensive role in the response of bryozoans to parasite infection. Serine proteases are one of the largest and diverse enzyme families and are involved in the activation of Toll signaling pathway [26]. Here, we found the upregulation of serine protease (6.03 fold), subtilisin-like serine protease-like protein PR1A (7.18 fold), transmembrane protease serine 9-like (9.26 fold), and chymotrypsin-like serine protease (11.63 fold) in the infected zooids. This suggests that parasite infection induces proteolysis activities, eventually leading to the activation of the toll-like receptor signaling pathway in bryozoans during the development of *T. bryosalmonae*. In support of this, we found upregulation of Toll signaling pathway genes such as TLR3 (2.59 fold), TLR4 (2.53 fold), and TLR13 (2.55 fold) in the infected zooids. Additionally, upregulation of TLRs such as TLR1 (4.1 fold), TLR13-like Chr1 (16.3 fold), and TLR19 (5.5 fold) has been reported in the kidney of brown trout during active *T. bryosalmonae* sporogenesis [23]. We presume that proteases are involved in numerous important processes in bryozoans and thereby play a role in the innate immune system in bryozoans during parasite infection.

Protease inhibitors are an enormously various and large group of proteins that inhibit the proteolytic activity of proteases in multiple physiological processes and are involved in the innate immune response [27]. We found upregulation of protease inhibitor-like protein (3.63 fold), fungal protease inhibitor-1-like (4.14 fold), and wound-induced proteinase inhibitor 1-like isoform X1 (7.33 fold) in the infected zooids. The upregulation of some protease inhibitors such as serine protease inhibitor, Kunitz-type protease inhibitor, and cysteine proteinase inhibitor protein have been reported in oligochaete worms and fish hosts in response to myxozoan parasites, *B. sowerbyi*, *Enteromyxum scophthalmi*, and *T. bryosalmonae* [18,22,28]. The upregulation of protease inhibitors may reflect a finely adapted innate immune defense system in bryozoans that is capable of monitoring self- and parasite-derived proteases and that may inactivate and eliminate the proteases involved in parasite invasion [29,30]. Proteases of myxozoans have been described as major virulence factors and are assumed to support the invasion of parasites into their hosts [31,32,33,34]. Similarly, proteases have been identified in *T. bryosalmonae* using RNA-seq analysis (authors own unpublished data). Consequently, the inhibition of parasite proteases is likely an important innate immune response employed by bryozoans in resisting *T. bryosalmonae* infection.

We found upregulation of other genes such as actins (actin alpha (10.42 fold), actin-1 (6.13 fold), actin binding protein (5.05 fold), actin depolymerizing protein (5.28 fold), and actin-related protein 2/3 complex subunit 1A (7.65 fold)) and heat shock proteins (HSPD1 (21.71 fold), HSPA2 (65.94 fold), HSP5 (3.33 fold), HSP60 (6.67 fold), HSP70 (61.84 fold), HSP70-3 (175.18 fold), HSP70A (12.23 fold), HSP71 (11.43 fold), HSP90 (47.08 fold), and HSP90B1 (5.70 fold)) in infected bryozoans. The upregulation of HSPs in oligochaete against *M. cultus* infection has been documented [18]. The roles played by actins and HSPs in bryozoans-parasite relationships is uncertain, however, it could be hypothesized that the relative upregulation in the transcription of actin genes may reflect overall enhancement in cellular motility and cell trafficking in infected bryozoans. HSPs may play a vital role in the repair process of the bryozoan cell by serving as molecular chaperones, thus suppressing or delaying cell necrosis during parasite development.

We also identified some other important downregulated genes in the infected zooids such as peptidoglycan-binding protein (−125.94 fold) and chitotriosidase-1 (−37.05 fold). Peptidoglycan-binding protein is required as a cell surface receptor for peptidoglycan elicitor signaling leading to innate immunity of the host [35]. Downregulation of peptidoglycan-binding protein in the infected bryozoans may suggest a restructuring of the protein in response to oxidizing conditions, preventing activation of the IMD signal transduction pathway following parasite infection [36]. The enzyme chitotriosidase-1 (CHIT1) is produced by activated macrophages and plays a role in immunity against the pathogens [37]. Zooids of bryozoans are made of either chitin or calcium carbonate. Chitin has been isolated and characterized from bryozoan *Plumatella repens* [38]. Hence, CHIT1 production is a significant factor in regulating the homeostasis by digesting the chitin. In this study, we observe that CHIT1 is significantly downregulated in bryozoans during *T. bryosalmonae* infection, suggesting that chitinase may be closely linked with inflammation and infection progression. However, the regulation and role of CHIT1 in bryozoans to parasite infection is not known.

## 4. Materials and Methods

### 4.1. Bryozoan F. sultana Colony

The colony of *F. sultana* was cultivated in our controlled laboratory conditions after hatching of specific pathogen-free (SPF) statoblasts. *F. sultana* colonies were maintained in an aquarium at 18 ± 1 °C and were fed with algae species mixtures (*Cryptomonas ovata*, *Synechococcus rubescens*, and *Synechococcus* spp.) according to Kumar et al. [39]. We cohabitated these colonies with *T. bryosalmonae*-infected brown trout. *F. sultana* colonies were maintained under optimal laboratory conditions according to Kumar et al. [39]. *F. sultana* colonies were observed using a dissecting microscope to check for the presence of *T. bryosalmonae*. Colonies cohabitated with *T. bryosalmonae*-infected brown trout showed the presence of parasite stages. However, no parasite stages were seen in SPF uninfected control *F. sultana* colonies. Additionally, these colonies were tested using PCR to confirm pathogen-free status [40].

The cohabitation of *F. sultana* colonies with infected brown trout was conducted in accordance with the relevant guidelines and regulations §26 of the Austrian Law for Animal Experiments, Tierversuchsgesetz 2012. The institutional ethics committee of the University of Veterinary Medicine, Vienna, Austria and the national authority approved this experiment under the permission numbers BMWFW GZ: 68.205/0181-WF/V/3b/2017 (23 October 2017) and BMWFW GZ: 2020-0.237.729 (17 April 2020).

### 4.2. Zooid Sampling

**Figure 9:**
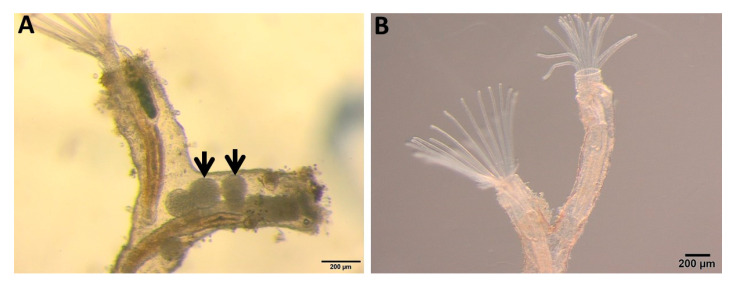
Bryozoan *Fredericella sultana* zooids. (**A**) Infected zooids showing parasite sacs (arrows) inside the body cavity. (**B**) Uninfected control zooids.

Zooids with overt infections (parasite sacs, Figure 9A), were collected from the infected *F. sultana* colony with the help of needles and forceps. Uninfected zooids (Figure 9B) were collected similarly. Zooids were immediately preserved in RNAlater and then stored at −80 °C for further use. Each sampled zooid includes a complete developed cystid body wall and polypide (the lophophore and digestive tract).

### 4.3. RNA Extraction

Total RNA was extracted from infected zooids (*n* = 3) and uninfected control zooids (*n* = 3) from RNAlater-preserved samples using the RNeasy Mini Kit (Qiagen, Hilden, Germany), utilizing the optional DNase digestion step. The quantity and integrity of the RNA were determined using a NanoDrop 200C spectrophotometer (Thermo, Waltham, MA, USA) and 4200 TapeStation using the RNA ScreenTape assay (Agilent, Santa Clara, CA, USA). RNA samples with an RNA integrity number above 8.0 were used for library preparation.

### 4.4. Library Construction and Sequencing

cDNA library was prepared from infected and uninfected control zooids of the three biological replicates. cDNA library construction, adaptor ligation, and library validation were carried out using the Illumina TruSeq RNA Sample Preparation Kit v2 (Illumina, San Diego, CA, USA) according to the manufacturer’s protocol. Illumina sequencing was performed on one lane of an Illumina NextSeq550 instrument implementing paired-end 150-bp reads.

### 4.5. RNA-Seq Data Analysis

Data analysis and quality filtering of the sequencing data was performed with CLC Genomics Workbench 12 software (Qiagen Bioinformatics, Aarhus, Denmark). Adapter sequences, low quality bases (Phred score ≤30) and reads shorter than 50 nt were removed. The filtered reads were mapped against the *F. sultana* transcriptome assembly (NCBI TSA accession: GHLZ01000000) [3] as a reference using the CLC Genomics RNA-seq tool with the default mapping parameters: mismatch cost 2, insertion cost 3, deletion cost 3, length fraction 0.5, and similarity fraction 0.8. Read counts were converted to transcripts per million (TPM) for normalization [41]. In the initial data exploration, we constructed a principal component analysis (PCA) and global Pearson correlation analysis to test whether both groups (infected and control) could be clustered separately. The analysis was performed in R directly and was based on the TPM normalized values of each sample.

Differential expression between infected and uninfected control zooid samples was analyzed using the ”exact test” for two-group comparisons [42]. All features with fold changes ≥2 or ≤−2 and FDR-adjusted *p*-values ≤ 0.01 were considered differentially expressed. For downstream analysis, orthologous human gene symbols obtained from the UniProt database (https://www.uniprot.org) were added to the *F. sultana* reference transcripts and genes that are represented by multiple contigs on the reference transcriptome were merged. For merged transcripts, the statistical results of the contig with the highest mean coverage (TPM) across all samples were used for further analysis assuming that more mapping reads represent more reliable expression data. A heatmap of the Z scaled resulting normalized TPM values of RNA-seq was generated using the heatmap.2 function of the gplots R package and hierarchical clustering was based on Euclidean distance and complete linkage.

### 4.6. Gene Ontology and Pathway Analysis

The list of DEGs was analyzed for significantly enriched Gene Ontology (GO) terms using the ClueGO v2.2.4 plugin of the biomolecular network software Cytoscape v3.1.1 [43]. Due to the lack of data for *F. sultana*, the analysis was based on human GO data. *p*-values ≤ 0.05 were considered significant. Additionally, Ingenuity Pathway Analysis (IPA; Qiagen Bioinformatics, Redwood City, CA, USA) was used to detect enriched canonical pathways in the list of DEGs. A *p*-value (Fisher’s exact test) ≤0.05 was considered significant. Activation z-scores calculated by IPA were used to predict the activation or inhibition of the networks. A z-score ≥2 denotes activation and a z-score ≤−2 denotes inhibition. PathVisio 3.3.0 [44] and GraphPad Prism 5 (GraphPad Software, San Diego, CA, USA) were used for visualization.

### 4.7. Validation of Differentially Expressed Genes

To confirm the RNA-seq results, gene expression levels were determined using the CFX96 Touch Real-Time PCR detection system (Bio-Rad, Hercules, CA, USA). cDNA was synthesized from total RNA as described above for infected (*n* = 6) and uninfected control (*n* = 6) samples using an iScript cDNA Synthesis Kit (Bio-Rad), according to the manufacturer’s protocol. Ten gene-specific primers (Table S6) were designed based on the assembled *F. sultana* transcriptome [3]. qPCR had a final volume of 20 μL, which contained 4 μL of 1:20 fold diluted cDNA, 0.5 μL of each primer (10 pmol/μL), 1X Universal SYBR Green Supermix Master Mix (Bio-Rad), and sterile DEPC-treated distilled water. qPCR condition was as follows: 94 °C for 5 min, and 94 °C for 30 s, 57–61 °C for 30 s, and 72 °C for 30 s for 37 cycles. A melting-point curve was then measured, starting from 57 °C and increasing by 0.5 °C every 10 s up to 95 °C, to detect any non-specific PCR products. All samples were analyzed in triplicate. Peptidyl-prolyl cis-trans isomerase, protein transport protein Sec61 subunit alpha, and ATP synthase subunit alpha were used as the reference genes. The expression of each target gene was normalized to the reference genes, calculated according to the 2−ΔΔCt method [45]. The statistical difference between infected and uninfected control samples was determined using the two-tailed unpaired Student’s t-test with Welch’s correction. Linear regression analysis was performed on corresponding log_2_ fold change values of RNA-seq and qRT-PCR to evaluate the relationship between them. For all statistical tests, a *p* value of <0.05 was regarded as significant and the data were analyzed in R statistical software version 3.5.3 [46].

## 5. Conclusions

We provide a global overview of the transcriptional responses of the bryozoan *F. sultana* to the parasitic malacosporean *T. bryosalmonae*. We found that *T. bryosalmonae* development caused dramatic alterations in gene regulation of *F. sultana*, leading to a sequence of cellular events that involve activating signaling modulation responses in *F. sultana*. Further analysis revealed that several well-known functional categories of genes and signaling pathways, which are associated with innate immune response, had been significantly enriched, including the functional categories of antigen presentation and processing, phagosome maturation, antioxidative responses, proteases, and proteases inhibitors. The transcriptome profile of *F. sultana* has yielded several novel putative innate immune system genes that may play critical roles in the response to *T. bryosalmonae*. This study improves our understanding of the molecular mechanisms underlying the interaction between *T. bryosalmonae* and their bryozoan host. The results of this study show a foundation for further analyses on bryozoan-responsive candidate genes, which would help us to understand bryozoan adaptation under *T. bryosalmonae* stress and eventually advantage future bryozoan molecular biology.

## Figures and Tables

**Table 1:** Sample description and numbers of RNA-seq reads. RNA-seq analysis of *F. sultana* zooids was performed using CLC Genomics Workbench 12 software.

Sample	Group	RNA Integrity Number	Number of Total Reads	Number of Trimmed Reads	Number of Mapping Reads
SPF_FS5	Uninfected control 1	10.0	47,040,144	42,441,722	27,015,844
SPF_FS6	Uninfected control 2	10.0	42,691,704	39,040,613	25,629,711
SPF_FS7	Uninfected control 3	10.0	41,354,820	37,161,830	23,951,516
FS1	Infected 1	8.7	45,873,568	42,174,572	21,607,226
FS2	Infected 2	10.0	47,279,902	43,698,337	17,195,568
FS3	Infected 3	9.8	49,668,044	44,862,085	18,654,732

**Table 2:** A brief summary of IPA analysis of DEGs identified in the infected zooids. The *p*-values were derived by IPA from Fisher’s exact test. Genes are rank ordered by fold change (positive or negative values). NaN, a z-score cannot be calculated for Ingenuity canonical pathways.

Top Canonical Pathways	−log (*p*-Value)	Ratio	z-Score
EIF2 Signaling	8.79 × 10^1^	4.05 × 10^−1^	6.949
Regulation of eIF4 and p70S6K Signaling	2.79 × 10^1^	2.44 × 10^−1^	0.816
mTOR Signaling	2.60 × 10^1^	1.99 × 10^−1^	0.905
Epithelial Adherens Junction Signaling	1.29 × 10^1^	1.63 × 10^−1^	NaN
Mitochondrial Dysfunction	1.18 × 10^1^	1.46 × 10^−1^	NaN
Phagosome Maturation	1.14 × 10^1^	1.55 × 10^−1^	NaN
Oxidative Phosphorylation	1.14 × 10^1^	1.83 × 10^−1^	3.578
Remodeling of Epithelial Adherens Junctions	1.09 × 10^1^	2.32 × 10^−1^	2.449
Unfolded protein response	1.01 × 10^1^	2.50 × 10^−1^	NaN
Sirtuin Signaling Pathway	8.06 × 10^0^	9.25 × 10^−2^	−0.943
Top Upstream Regulators	*p*-value of overlap	Activation State	Activation z-Score
MYCN	1.15 × 10^−62^	Activated	6.655
RICTOR	1.57 × 10^−46^	Inhibited	−7.693
MYC	3.66 × 10^−22^	Activated	5.946
TCR	1.23 × 10^−21^	Activated	4.499
APP	1.84 × 10^−20^	Activated	3.069
LONP1	6.62 × 10^−13^	Inhibited	−2.813
HSP90B1	1.32 × 10^−12^	Activated	3.0
NFE2L2	1.14 × 10^−11^	Activated	4.073
DAP3	2.23 × 10^−10^	Activated	2.121
INSR	1.43 × 10^−9^	Activated	2.554
Top Upregulated Genes	Fold Change	Top Downregulated Genes	Fold Change
*PPIA*	606.04	*LYM3*	−125.94
*HSPA1A*	473.11	*ADGRG6*	−59.52
*PRPF8*	256.84	*MRC1*	−58.20
*PPIL1*	242.79	*LEC6*	−46.43
*SF3B4*	232.08	*PLCL*	−42.01
*TUBB*	194.51	*LECG*	−39.98
*HSP70-3*	175.18	*CHIT1*	−37.05
*PPP2CB*	169.71	*ADF1*	−34.09
*HIST1H2AG*	161.56	*AGRG7*	−30.27
*CALM1*	146.60	*RL30E*	–21.67

## Supplementary Materials

Supplementary materials can be found at https://www.mdpi.com/1422-0067/21/16/5910/s1. Table S1: List of all significant differentially up- and downregulated genes of infected zooids, Table S2: Enriched gene ontology terms of all differentially up- and downregulated genes of infected zooids, Table S3: List of enriched canonical pathways, Table S4: List of predicted upstream regulators, Table S5: Enriched gene ontology terms of all differentially upregulated genes of infected zooids, Table S6: List of quantitative real-time PCR primers.

## Abbreviations

BLAST	Basic Local Alignment Search Tool
TPM	Transcripts per million
NCBI	National Center for Biotechnology Information
qRT PCR	Quantitative real time PCR
